# Texas Lifestyle Limits Transmission of Dengue Virus

**DOI:** 10.3201/eid0901.020220

**Published:** 2003-01

**Authors:** Paul Reiter, Sarah Lathrop, Michel Bunning, Brad Biggerstaff, Daniel Singer, Tejpratap Tiwari, Laura Baber, Manuel Amador, Jaime Thirion, Jack Hayes, Calixto Seca, Jorge Mendez, Bernardo Ramirez, Jerome Robinson, Julie Rawlings, Vance Vorndam, Stephen Waterman, Duane Gubler, Gary Clark, Edward Hayes

**Affiliations:** * Centers for Disease Control and Prevention, Fort Collins, Colorado, USA; † City of Laredo Health Department, Laredo, Texas, USA; ‡ Secretaria de Salud, Mexico City, Mexico; § University of Texas, Boerne, Texas, USA; ¶ Texas Department of Health, Laredo, Texas, USA; # Secretaria de Salud de Tamaulipas, Nuevo Laredo, Tamaulipas, Mexico

**Keywords:** *Aedes aegypti*, air-conditioning, arboviruses, climate change, dengue fever, epidemiology, global warming, human behavior, mosquito behavior, research

## Abstract

Urban dengue is common in most countries of the Americas, but has been rare in the United States for more than half a century. In 1999 we investigated an outbreak of the disease that affected Nuevo Laredo, Tamaulipas, Mexico, and Laredo, Texas, United States, contiguous cities that straddle the international border. The incidence of recent cases, indicated by immunoglobulin M antibody serosurvey, was higher in Nuevo Laredo, although the vector, *Aedes aegypti*, was more abundant in Laredo. Environmental factors that affect contact with mosquitoes, such as air-conditioning and human behavior, appear to account for this paradox. We conclude that the low prevalence of dengue in the United States is primarily due to economic, rather than climatic, factors.

Outbreaks of mosquito-borne disease are commonly assumed to occur wherever competent vectors and a suitable climate exist, and that “global warming”—climate change caused by human activities—will cause these diseases to move to higher altitudes and latitudes. In many parts of the world, however, such diseases have become uncommon, despite an abundance of vectors and an ideal climate.

Denguelike illness was first noted in the New World as a major outbreak in Philadelphia in 1780 (*1*), and similar episodes occurred in the United States for more than 150 years. In 1922, the disease struck many major cities in the southern states, including an estimated 500,000 cases in Texas. Another widespread outbreak occurred in 1947–48 (*2*). In the past 50 years, however, autochthonous cases have been rare, despite an abundance of *Aedes aegypti* in the southeastern United States, and the arrival of millions of travelers from neighboring countries where the disease is endemic. From 1980 to 1999, only 64 locally acquired cases were confirmed in Texas, whereas 62,514 suspected cases were recorded in three adjoining Mexican states—Coahuila, Nuevo León, and Tamaulipas. In the same period, immigration authorities reported < 70 million personal crossings from these states into Texas in a single year (*3*). Thus, the international border separates a dengue-endemic region from one in which the disease is rare.

Laredo, Texas, United States (population 200,000), and Nuevo Laredo, Taumalipas, Mexico (population 289,000), are essentially a single city (locally known as “los dos Laredos”) divided by a small river, the Rio Grande (Figure). The rapid growth of this metropolitan area—70% in the past decade—is mainly due to massive cross-border traffic across three multilane bridges (Laredo Chamber of Commerce. Laredo, Texas; available from: URL: http://www.laredochamber.com/contact-information.htm). In the summer of 1999, toward the end of a local dengue outbreak, we conducted a seroepidemiologic survey to examine factors affecting dengue transmission on both sides of the border.

**Figure:**
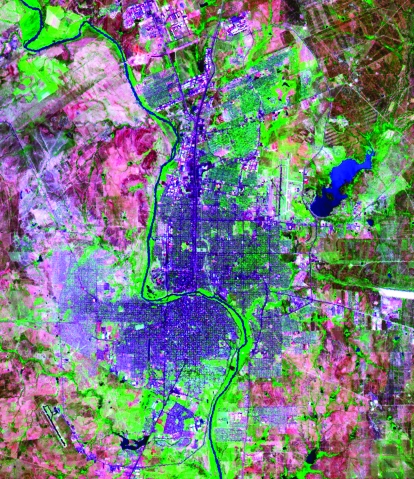
Laredo, Texas (east of the Rio Grande river) and Nuevo Laredo, Taumalipas (west of the river). Blue, water; green, vegetation; blue-violet, roads and buildings; blue-violet areas on the western side of Nuevo Laredo, low-income neighborhoods; pink, land with little or no vegetation. (National Aeronautics and Space Administration (NASA)/U.S. Geological Survey LANDSAT 7 image [TM bands 7, 4, and 3];courtesy of NASA.)

## Methods

Households were selected by a modified version of the cluster survey of the World Health Organization Expanded Program on Immunization (*4*). First, we mapped the population of each census block in Laredo and in a major portion of Nuevo Laredo (Sector 1). In each city, 30 clusters were chosen from these census blocks by using a selection probability proportional to population. Four city blocks were randomly chosen from each of these clusters, and individual houses in one or more of those blocks were selected at random (where block maps were available) or systematically from a randomly chosen starting point. Blocks were sampled until 7–12 households had been enrolled from each cluster.

Binational teams, each composed of an epidemiologist, a nurse, and an entomologist, conducted the surveys. A blood sample was obtained by fingerstick from a randomly selected resident (ages 18–65)^1^. A short questionnaire solicited general household information (number of inhabitants, type of construction, proximity to neighboring houses, number of bedrooms, presence and type of air-cooling system, and the presence and quality of window screens). Demographic data and travel histories of the blood donors were also recorded. Yards and patios were searched for *Ae. aegypti* breeding sites.

Serum samples were tested for anti-dengue immunoglobulin M (IgM) by IgM antibody-capture enzyme-linked immunosorbent assay (MAC-ELISA), and for anti-dengue IgG by IgG-ELISA and mixed dengue antigens (*5*,*6*). Data were analyzed with SAS v.6.12 (SAS Institute, Inc., Cary, NC) and SAS-callable SUDAAN (Research Triangle Institute, Research Triangle Park, NC) software. Risk factors for IgM and IgG seropositivity were assessed by multivariable weighted logistic regression, accounting for stratification by country, clustering within each city, and different numbers of surveys per cluster. Backward selection of variables was used to create the final models. Variables were retained if statistically significant (p<0.05).

## Results

Surveys were completed in 622 households (309 in Laredo, 313 in Nuevo Laredo), and 516 persons (228 in Laredo, 288 in Nuevo Laredo) provided blood samples. IgM seropositivity (Table 1) was lower in Laredo (1.3%; 95% confidence interval [CI] 0 to 3%) than in Nuevo Laredo (16%; CI 12% to 20%). IgG seropositivity (Table 1) was also lower in Laredo (23%; CI 17% to 28% vs. 48%; CI 41% to 55%). Conversely, mosquito-infested containers were more abundant on the Texas side of the border: the Breteau Index (the number of infested containers per 100 houses) was 91 in Laredo versus 37 in Nuevo Laredo. Eighty-two percent of homes in Laredo had central or room air-conditioning versus 24% in Nuevo Laredo. In Laredo, evaporative coolers (a low-technology air-conditioning device that cools and humidifies air by drawing it from outdoors through a continually wetted screen) were less prevalent, a greater proportion of houses had intact screens, the average distance between houses was greater, and fewer persons lived in each house (Table 2).

**Table 1:** Seroprevalence of anti-dengue immunoglobulin G (IgG) and IgM antibodies in Nuevo Laredo, Mexico, and Laredo, Texas, United States

	**Nuevo Laredo**	**Laredo**
**Households**	**313**	**309**
**Serum samples**	**288**	**228**
**IgM prevalence**	**16% (12 to 20)^a^**	**1.3% (0 to 3)^a^**
**IgG prevalence**	**48% (41 to 55)^a^**	**23% (17 to 28)^a^**

^a^Weighted point estimate and (95% confidence interval).

**Table 2:** Selected housing and travel characteristics in Nuevo Laredo and Laredo residents

**Characteristic**	**Nuevo Laredo**	**Laredo**
**Central air-conditioning**	**2%**	**36%**
**Room air conditioner**	**23%**	**52%**
**Evaporative cooler**	**29%**	**17%**
**Screens on windows**	**54%**	**78%**
**Intact window screens**	**36%**	**60%**
**Mean no. occupants/residence**	**4.5**	**3.8**
**Mean distance to neighbors (m)**	**3.0**	**4.5**
**No travel outside Laredo/Nuevo Laredo area**	**70%**	**63%**
**Crossed border within 3 months of survey**	**52%**	**43%**

Univariate analysis indicated a significant association between IgM seropositivity and five variables: absence of air-conditioning, fewer room air-conditioning units, the presence of an evaporative cooler, no travel outside the Laredo/Nuevo Laredo area, and shorter distances to neighboring houses (Table 3). IgG seropositivity was significantly associated with absence of central air-conditioning, fewer room air-conditioning units, smaller plot size, and a shorter distance to neighboring houses (Table 4).

**Table 3:** Risk factors associated with anti-dengue immunoglobulin M (IgM) seropositivity in Nuevo Laredo/Laredo residents, by univariate analysis

**Risk factor**	**Prevalence ratio**	**Mean value for IgM seropositive residents**	**Mean value for IgM seronegative residents**	**(95% confidence interval) or** **p value**
**No air-conditioning**	**2.6**	**—**	**—**	**(1.3 to 5.2)**
**Evaporative cooler**	**2**	**—**	**—**	**(1.2 to 3.3)**
**No travel beyond Laredo/Nuevo Laredo area**	**1.9**	**—**	**—**	**(1.0 to 3.5)**
**No. room air conditioners**	**—**	**0.3**	**0.6**	**p=0.05**
**Distance to neighbor (m)**	**—**	**2.4**	**3.8**	**p=0.003**

**Table 4:** Risk factors associated with anti-dengue IgG seropositivity in Nuevo Laredo/Laredo residents, by univariate analysis

**Factor**	**Prevalence ratio**	**Mean value for IgG seropositive residents**	**Mean value for IgG seronegative residents**	**(95% confidence interval) or p value**
**No air-conditioning**	**1.65**	**—**	**—**	**(1.27 to 2.15)**
**No. occupants**	**—**	**4.7**	**4.3**	**p=0.05**
**Lot size (m^2^)**	**—**	**377**	**395**	**p=0.03**
**No. room air conditioners**	**—**	**0.4**	**0.7**	**p=0.002**
**Distance to neighbor (m)**	**—**	**3.3**	**3.9**	**p=0.03**

On multivariate analysis, backward selection of variables yielded two that remained significantly associated with IgM seropositivity: absence of air-conditioning (odds ratio [OR] 2.6; CI 1.2 to 5.6) and no history of travel beyond Laredo/Nuevo Laredo in the previous 3 months (OR 2.0; CI 1.0 to 4.0). IgG seropositivity was associated with absence of air-conditioning (OR 2.4; CI 1.5 to 4.0), a history of crossing the border during the previous 3 months (OR 1.8; CI 1.1 to 2.8), and a greater number of occupants per household (OR 1.1; CI 1.0 to 1.2). By using the calculated prevalence ratio of 2.6 as an estimate of the relative risk of dengue in houses without air-conditioning, the proportion of dengue infections attributable to lack of air-conditioning in Nuevo Laredo was 55%, i.e., 55% of cases of dengue in Nuevo Laredo would not have occurred if all households in Nuevo Laredo had air-conditioning.

## Discussion

Given the proximity of the two cities, the difference in transmission rates cannot be attributed to climate. Moreover, the mean daily temperature for August, the peak month of transmission, was 32.2°C (mean maximum 40.0°C; mean minimum 24.4°C), several degrees higher than the mean for the hottest months on Caribbean islands where dengue is common. Indeed, summer temperatures throughout the range of *Ae. aegypti* in the southern United States are hotter than in many tropical regions where the disease is endemic.

Despite mosquito control campaigns on both sides of the border, *Ae. aegypti* infestation rates in Laredo were remarkably high. The Breteau Index was on a par with that observed during major dengue epidemics in Puerto Rico (CDC, unpub. data). The House Index (the percentage of houses with at least one infested container) was 37%, seven times higher than the level (5%) equated with a “high risk” of dengue transmission by the World Health Organization (*7*). Thus, vector populations cannot account for the low rate of transmission on the Texas side of the border.

*Ae. aegypti* is closely associated with human habitation and readily enters buildings to feed and to rest during periods of inactivity (*8*). In this context, casual observation supported the association of lack of air-conditioning with dengue transmission. In Laredo, most shops, restaurants and other public places are air conditioned and have closed windows and self-closing doors, as do houses in residential areas, even in low income neighborhoods. By contrast, in Nuevo Laredo, many shops, bars, and restaurants are open to the street, and the windows and doors of houses are left open, particularly in the daytime. Thus, there is less opportunity for mosquito/human contact in Laredo than in the Mexican city.

More than 85% of all buildings in Texas are fully air conditioned (American Society of Heating, Refrigerating and Air-Conditioning Engineers, Inc., Atlanta, GA; available from: URL: http://www.ashrae.org/). Indeed, air-conditioning is ubiquitous in many parts of the United States. To maximize heating/cooling efficiency, windows are usually fully glazed and are often kept permanently closed. Thus, most people spend much of their daily life sequestered in sealed buildings. Even if infected mosquitoes gain entry to such buildings, the artificially dry atmosphere lowers their survival rate, and the cool temperature extends the extrinsic incubation period, reducing the likelihood of transmission. Presumably, when denied access to humans, mosquitoes must seek other hosts. In Puerto Rico and Thailand, some *Ae. aegypti* feed on dogs, even when humans are readily accessible (*9*,*10*). In Laredo, we observed that large dogs were housed in outdoor kennels at many homes. Whether these animals are an important blood source for the species would be an interesting topic for future research.

The dollar cost of electricity is similar in Laredo and Nuevo Laredo, but income, as indicated by per capita gross domestic product, is much higher in Texas than in Taumalipas (Table 5). The proportional cost of maintaining air-conditioning for an entire dengue season is therefore much higher for the average family in Mexico and is unaffordable for the majority. Thus, the ultimate determinant of dengue prevalence in this setting is socioeconomic rather than environmental.

**Table 5:** Estimated cost (US dollars) of air-conditioning a house in Texas vs. Taumalipas, Mexico

	**Texas**	**Taumalipas**
**Cost per kilowatt hour (kWh)**	**0.06119**	**0.04863**
**Cost of 25,000 kWh**	**1,530**	**1,216**
**Per capita GDP^a^**	**34,288**	**5,014**
**Percentage of per capita GDP**	**4.5**	**24.2**

^a^GDP, gross domestic product.

## Conclusion

It has frequently been stated that dengue, malaria, and other mosquito-borne diseases will become common in the United States as a result of global warming (*11*–*14*). Such predictions often refer to vectorial capacity, a simple model that incorporates the population density, biting frequency, and daily survival probability of the vector, and the extrinsic incubation period of the pathogen (*15*,*16*). Although the vectorial capacity model has proved useful for interpreting entomo-epidemiologic data, particularly for transmission of malaria (*17*), it does not incorporate factors like air-conditioning, use of vaporative coolers, and the behavior of mosquitoes and humans. If the current warming trend in world climates continues, air-conditioning may become even more prevalent in the United States, in which case, the probability of dengue transmission is likely to decrease. If the economy of Mexico continues to grow, the use of air-conditioners may gain momentum south of the border.

## Footnotes

1 The investigation plan was reviewed by the Human Subjects Coordinator at the National Center for Infectious Diseases, Centers for Disease Control and Prevention, and determined to be a public health response that did not require further human subjects review. Blood samples were taken only from consenting adults.
